# *TLR4* 896A/G and *TLR9* 1174G/A polymorphisms are associated with the risk of infectious mononucleosis

**DOI:** 10.1038/s41598-020-70129-4

**Published:** 2020-08-04

**Authors:** Agnieszka Jabłońska, Mirosława Studzińska, Leszek Szenborn, Małgorzata Wiśniewska-Ligier, Monika Karlikowska-Skwarnik, Tomasz Gęsicki, Edyta Paradowska

**Affiliations:** 1 Laboratory of Virology, Institute of Medical Biology, Polish Academy of Sciences, Lodz, Poland; 2 Department of Pediatric Infectious Diseases, Wroclaw Medical University, Wroclaw, Poland; 3 Department of Pediatrics, Immunology, and Nephrology, Polish Mother’s Memorial Hospital Research Institute, Lodz, Poland; 4 Department of Otolaryngology, Polish Mother’s Memorial Hospital, Research Institute, Lodz, Poland

**Keywords:** Virology, Infectious diseases, Pattern recognition receptors

## Abstract

Toll-like receptors (TLRs) recognize pathogen-associated molecular patterns and activate innate and adaptive immune responses. Single nucleotide polymorphisms (SNPs) within the *TLR* genes may influence host–pathogen interactions and can have an impact on the progression of infectious diseases. The present study aimed to investigate the genotype distribution of *TLR2* (2029C/T, rs121917864; 2258G/A, rs5743708), *TLR4* (896A/G, rs4986790), and *TLR9* (− 1237T/C, rs5743836; − 1486T/C, rs187084; 1174G/A, rs352139; and 2848C/T, rs352140) polymorphisms in 149 children and adolescents with infectious mononucleosis (IM) and 140 healthy individuals. The potential association of *TLR* SNPs with the clinical manifestations of EBV infection was also studied. The presence of *TLR2*, *TLR4*, and *TLR9* SNPs was identified by polymerase chain reaction–restriction fragment length polymorphism (PCR–RFLP). EBV DNA loads were detected by quantitative real-time PCR assay. The *TLR4* 896 GG and the *TLR9* 1174 GA genotypes were associated with an increased risk of EBV-related IM in examined patients (*p* = 0.014 and *p* = 0.001, respectively). The heterozygous genotype of the *TLR4* 896A/G SNP was associated with an increased risk of elevated liver enzyme levels and leukocytosis (*p* < 0.05). Our preliminary study revealed that the *TLR4* 896A/G and the *TLR9* 1174G/A polymorphisms seem to be related to the course of acute EBV infection in children and adolescents.

## Introduction

Epstein–Barr virus (EBV) is an oncogenic, human γ-herpesvirus and one of the most ubiquitous pathogens, infecting more than 90% of the adult population worldwide^1,2^. Primary EBV infection is usually asymptomatic and occurs during infancy or childhood. In developing countries, however, primary EBV infection is often delayed until adolescence and early adulthood and it occurs as infectious mononucleosis (IM). It is a self-limiting disease characterized by fatigue, fever, headache, lymphadenopathy, exudative pharyngitis, enlarged spleen and liver, in laboratory findings with elevated activity of the liver enzyme in serum and the occurrence of atypical-lymphocytes in blood smear^1,3,4^. After primary infection, EBV becomes latent within the B lymphocytes and epithelial cells^5,6^. The virus establishes lifelong persistent infection in normal immunocompetent hosts as well as it is able to be reactivated for spreading to new hosts. Reactivated from latency EBV may be associated with the development of a wide spectrum of malignancies such as Burkitt’s lymphoma (BL), Hodgkin and non-Hodgkin lymphomas (NHL), posttransplant lymphoproliferative disorder (PTLD), as well as epithelial tumors such as nasopharyngeal and gastric carcinomas^7,8^. An association between infectious mononucleosis and an increased risk of classical Hodgkin lymphoma in young adults was observed^9,10^.

Toll-like receptors (TLRs) are a family of transmembrane, evolutionarily conserved receptors that recognize molecular patterns unique to pathogens and activate host innate immune response^11^. The innate immune response to EBV is initiated after the recognition of its major envelope glycoprotein gp350/220 by TLR2 or activation of complement receptors CR2/CD21 and/or CR1/CD35 on B cells^12–15^. In addition, EBV-encoded non-structural protein dUTPase, expressed during the lytic cycle of viral replication, activates the nuclear factor кB (NF-кB) via adaptor myeloid differentiated 88 (MyD88)-dependent signaling cascade and induces expression of proinflammatory cytokines in macrophages^16^. In plasmacytoid dendritic cells (pDCs) and primary monocytes, infection of EBV activates TLR7 and/or TLR9 signaling pathway leading to interferon (IFN)-α and IFN-γ production^17–19^. Although many studies on TLRs in EBV-associated malignancies have been carried out in the past years, little is known about the role of TLRs in the pathogenesis of IM in humans. Rare single *TLRs* mutations have been associated with increased susceptibility to herpesvirus infections^20–22^. Understanding the genetic basis of susceptibility to EBV infections is important to the development of antiviral strategies. In the present study, we have assessed the distribution of the seven single nucleotide polymorphisms (SNPs) in the *TLR2* (2029C/T, 2258G/A), the *TLR4* (896A/G), and the *TLR9* (− 1237T/C, − 1486T/C, 1174G/A, and 2848C/T) genes in children and adolescents with acute EBV infection.

## Results

### Clinical outcome assessment

At the time of this study 149 participants with acute IM, including 93 children (median age: 4.3 years; range 3 months–9.0 years) and 56 adolescents (median age: 14.2 years; range 10.0 years–17.5 years), and 140 healthy volunteers were examined. In all patients with IM, EBV infection was confirmed by the serologic and/or virologic tests. In brief, EBV-IgM was positive for 109 individuals, viral capsid antigen (VCA)-IgG for 46, EBV-IgG for 71, and EBNA-IgG was positive for 30 participants. Almost all patients (38/40) in whom specific IgM was undetected were children < 7 years of age. The results are presented separately for the two patient groups (children and adolescents) in Table 1. Higher frequency of EBV-specific IgM in adolescents than children (*p* = 0.008) was found, whereas children presented higher levels of the EBNA IgG antibodies (*p* = 0.037). The EBV DNA was detected in whole blood samples from 69 (74.2%) children and 35 (62.5%) adolescents (mean 2.13 × 10^4^ copies/ml ± 1.23 × 10^5^ copies/ml and mean 1.20 × 10^4^ copies/ml ± 4.74 × 10^4^ copies/ml, respectively). In all patients with IM, the presence of the EBV DNA in mouth saliva and/or throat swabs was found (mouth saliva: mean 8.77 × 10^5^ copies/ml ± 1.75 × 10^6^ copies/ml; throat swabs: mean 5.55 × 10^4^ copies/ml ± 2.23 × 10^5^ copies/ml). Patients were classified as having clinically confirmed IM when the following signs or symptoms were observed: atypical-lymphocytes, pharyngitis, lymphadenopathy, fever, fatigue, and enlarged spleen and/or liver. Clinical manifestations of acute IM were found in all examined children and youth patients. However, as expected from other studies, specific signs and symptoms of IM (elevated liver enzyme levels, atypical-lymphocytes, and thrombocytopenia) were detected more frequently in adolescents than in children (Table 1). It was found that elevated liver enzyme levels were more common in adolescents than children (82.1% vs. 61.3%, *p* = 0.010). There were also more adolescents than children with atypical-lymphocytes (66.1% vs. 48.4%, *p* = 0.042) and thrombocytopenia (42.9% vs. 19.4%, *p* = 0.003). In contrast, incidents of amoxicillin rash were observed more frequently among children than adolescents (25.8% vs. 10.7%, *p* = 0.034).

**Table 1:** Clinical features of patients with infectious mononucleosis.

Clinical feature	Number of IM patients, n (%)		*p*-value	Total, n (%)
	Children	Adolescents		
**Gender**				
Male	62/93 (66.7)	23/56 (41.1)	**0.004**	85/149 (57.0)
Female	31/93 (33.3)	33/56 (58.9)	**0.004**	64/149 (43.0)
**Symptoms**				
Eyelid edema	7/93 (7.5)	9/56 (16.1)	0.111	16/149 (10.7)
Fever	65/93 (69.9)	43/56 (76.8)	0.450	108/149 (72.5)
Hepatomegaly	36/93 (38.7)	24/56 (42.9)	0.730	60/149 (40.3)
Lymphadenopathy	59/93 (63.4)	37/56 (66.1)	0.860	96/149 (64.4)
Pharyngitis	62/93 (66.7)	36/56 (64.3)	0.859	98/149 (65.8)
Rash	24/93 (25.8)	6/56 (10.7)	**0.034**	30/149 (20.1)
Splenomegaly	38/93 (40.9)	24/56 (42.9)	0.865	62/149 (41.6)
Vomiting, diarrhea	7/93 (7.5)	3/56 (5.4)	0.744	10/149 (6.7)
**Laboratory abnormalities**				
Atypical-lymphocytes	45/93 (48.4)	37/56 (66.1)	**0.042**	82/149 (55.0)
Elevated liver enzyme level	57/93 (61.3)	46/56 (82.1)	**0.010**	103/149 (69.1)
Leukocytosis	47/93 (50.5)	35/56 (62.5)	0.176	82/149 (55.0)
Thrombocytopenia	18/93 (19.4)	24/56 (42.9)	**0.003**	42/149 (28.2)
**Anti-EBV serologic status**				
EBV-IgM positive	61/93 (65.6)	48/56 (85.7)	**0.008**	109/149 (73.2)
VCA-IgG positive	37/55 (67.3)	9/11 (81.8)	0.482	46/66 (69.7)
EBV-IgG positive	31/38 (81.6)	40/45 (88.9)	0.368	71/83 (85.5)
EBNA-IgG positive	23/93 (24.7)	7/56 (12.5)	**0.037**	30/149 (20.1)

Significant *p*-values were highlighted in bold.

*IM* infectious mononucleosis, *n* number of patients, *p* Fisher’s exact test.

### The heterozygous genotype of the *TLR9* 1174G/A SNP occurs more frequently in patients with IM

The *TLR2* 2029C/T, 2258G/A, *TLR4* 896A/G, and *TLR9* − 1237T/C, − 1486T/C, 1174G/A, and 2848C/T SNPs were genotyped in 149 patients with IM and 140 healthy volunteers (Table 2). For the *TLR2* 2029C/T SNP and the *TLR4* 896A/G SNP, the homozygous recessive genotypes were detected more frequently in children and adolescents with IM than in healthy subjects (4.0% *vs.* 0.0%, *p* = 0.030 and 6.7% *vs.* 0.7%, *p* = 0.011, respectively; Table 2). Consequently, the wild-type C alleles of these SNPs were detected more frequently in uninfected individuals compared with EBV-infected cases (*p* = 0.039 and *p* = 0.004, respectively; Table 2). The heterozygous genotype of the *TLR9* 1174G/A polymorphism was more common in patients with IM than in healthy individuals (47.0% vs. 30.0%, *p* = 0.004), while no difference in the frequency of the alleles was observed (*p* > 0.05). Mutation present in at least one allele of the *TLR9* 2848C/T SNP occurred more frequently in patients with IM than in healthy subjects (55.4% vs. 38.9%, *p* = 0.0001; Table 2). No significant differences for the *TLR2* 2258G/A and the *TLR9* − 1237T/C and − 1486C/T SNPs were found. Moreover, no sex differences in the *TLR* SNPs frequency among patients with IM were observed. The expected genotype frequencies for *TLR9* − 1486T/C and 2848C/T SNPs were not in Hardy–Weinberg Equilibrium (HWE; *p* < 0.001 for both) and were excluded from further analysis.

**Table 2:** Frequencies of *TLR2*, *TLR4*, and *TLR9* SNPs genotypes and alleles in individuals with EBV-related infectious mononucleosis.

*TLR* SNP	Genotype/Allele	Patients with IM, n (%)	Healthy volunteers, n (%)	*p*-value
*TLR2* 2029C/T	CC	92 (61.8)	100 (71.4)	0.105
	CT	51 (34.2)	40 (28.6)	0.314
	TT	6 (4.0)	0 (0.0)	**0.030**
	C	235 (78.9)	240 (85.7)	**0.039**
	T	63 (21.1)	40 (14.3)	**0.039**
*TLR2* 2258G/A	GG	125 (83.9)	113 (80.7)	0.538
	GA	19 (12.7)	27 (19.3)	0.149
	AA	5 (3.4)	0 (0.0)	0.061
	G	269 (90.3)	253 (90.4)	1.000
	A	29 (9.7)	27 (9.6)	1.000
*TLR4* 896A/G	AA	128 (85.9)	130 (92.9)	0.060
	AG	11 (7.4)	9 (6.4)	0.819
	GG	10 (6.7)	1 (0.7)	**0.011**
	A	267 (89.6)	269 (96.1)	**0.004**
	G	31 (10.4)	11 (3.9)	**0.004**
*TLR9* − 1237T/C	TT	146 (98.0)	140 (100.0)	0.248
	TC	2 (1.3)	0 (0.0)	0.499
	CC	1 (0.7)	0 (0.0)	1.000
	T	294 (98.7)	280 (100.0)	0.124
	C	4 (1.3)	0 (0.0)	0.124
*TLR9* − 1486T/C	TT	56 (37.6)	63 (45.0)	0.232
	TC	59 (39.6)	45 (32.1)	0.220
	CC	34 (22.8)	32 (22.9)	1.000
	T	171 (57.4)	171 (61.1)	0.397
	C	127 (42.6)	109 (38.9)	0.397
*TLR9* 1174G/A	GG	77 (51.7)	88 (62.9)	0.058
	GA	70 (47.0)	42 (30.0)	**0.004**
	AA	2 (1.3)	10 (7.1)	**0.017**
	G	224 (75.2)	218 (77.9)	0.493
	A	74 (24.8)	62 (22.1)	0.493
*TLR9* 2848C/T	CC	37 (24.8)	64 (45.7)	**0.0002**
	CT	59 (39.6)	43 (30.7)	0.139
	TT	53 (35.6)	33 (23.6)	**0.029**
	C	133 (44.6)	171 (61.1)	**0.0001**
	T	165 (55.4)	109 (38.9)	**0.0001**

Significant *p*-values were highlighted in bold.

*IM* infectious mononucleosis, *n* number of cases, *p* Fisher’s exact test.

### The *TLR4* 896 GG and the *TLR9* 1174 GA genotypes are associated with increased risk of IM

The *TLR4* 896 GG genotype was associated with a tenfold increased risk of IM (OR 10.00; 95% CI 1.26–79.14; *p* = 0.004, in the recessive model; Table 3). This SNP showed a higher risk of IM even after Bonferroni correction for multiple testing (*p*_*B*_ = 0.01). A higher risk of IM in patients with the *TLR9* 1174 GA genotype was also found (OR 1.90; 95% CI 1.17–3.11; *p* = 0.001, in the codominant model), although a lower incidence of IM in patients with AA genotype was observed (OR 0.18; 95% CI 0.04–0.82; *p* = 0.010, in the recessive model). In addition, IM patients with the heterozygous genotype of the *TLR2* 2029C/T had a slightly increased risk of the disease compared to healthy individuals (OR 1.39; 95% CI 0.84–2.29; *p* = 0.008, in the codominant model).

**Table 3:** The distribution of genotypes frequencies of *TLR2*, *TLR4*, and *TLR9* SNPs in children and adolescents, and the relationship between polymorphisms and the risk of infectious mononucleosis.

*TLR* SNPs	Model	Genotype	Genotype frequencies, n (%)^a^		Unadjusted		Adjusted^b^	
			Patients with IM	Healthy subjects	OR (95% CI)	*p*-value	OR (95% CI)	*p*-value
*TLR2* 2029C/T	Codominant	CC	92 (61.8)	100 (71.4)	1.00	**0.008**	1.00	0.018
		CT	51 (34.2)	40 (28.6)	1.39 (0.84–2.29)		2.63 (1.32–5.25)	
		TT	6 (4.0)	0 (0.0)	NA (0.00–NA)		NA (0.00–NA)	
	Dominant	CC	92 (61.8)	100 (71.4)	1.00	0.081	1.00	**0.006**
		CT-TT	57 (38.2)	40 (28.6)	1.55 (0.95–2.54)		2.66 (1.33–5.30)	
	Recessive	CC-CT	143 (96.0)	140 (100.0)	1.00	**0.005**	1.00	0.5
		TT	6 (4.0)	0 (0.0)	NA (0.00–NA)		NA (0.00–NA)	
	Overdominant	CC-TT	98 (65.8)	100 (71.4)	1.00	0.3	1.00	**0.007**
		CT	51 (34.2)	40 (28.6)	1.30 (0.79–2.14)		2.59 (1.30–5.17)	
*TLR2* 2258G/A	Codominant	GG	125 (83.9)	113 (80.7)	1.00	0.013	1.00	0.71
		GA	19 (12.8)	27 (19.3)	0.64 (0.34–1.21)		0.68 (0.26–1.74)	
		AA	5 (3.4)	0 (0.0)	NA (0.00–NA)		NA (0.00–NA)	
	Dominant	GG	125 (83.9)	113 (80.7)	1.00	0.48	1.00	0.41
		GA-AA	24 (16.1)	27 (19.3)	0.80 (0.44–1.47)		0.68 (0.26–1.74)	
	Recessive	GG-GA	144 (96.6)	140 (100.0)	1.00	**0.01**	1.00	0.97
		AA	5 (3.4)	0 (0.0)	NA (0.00–NA)		NA (0.00–NA)	
	Overdominant	GG-AA	130 (87.2)	113 (80.7)	1.00	0.13	1.00	0.41
		GA	19 (12.8)	27 (19.3)	0.61 (0.32–1.16)		0.68 (0.26–1.74)	
*TLR4* 896A/G	Codominant	AA	128 (85.9)	130 (92.9)	1.00	0.014	1.00	**0.003**
		AG	11 (7.4)	9 (6.4)	1.24 (0.50–3.10)		0.00 (0.00–NA)	
		GG	10 (6.7)	1 (0.7)	10.16 (1.28–80.46)		13.00 (1.41–119.62)	
	Dominant	AA	128 (85.9)	130 (92.9)	1.00	0.054	1.00	0.68
		AG-GG	21 (14.1)	10 (7.1)	2.13 (0.97–4.71)		1.30 (0.39–4.37)	
	Recessive	AA-AG	139 (93.3)	139 (99.3)	1.00	**0.004**	1.00	**0.008**
		GG	10 (6.7)	1 (0.7)	10.00 (1.26–79.14)		13.89 (1.51–127.78)	
	Overdominant	AA-GG	138 (92.6)	131 (93.6)	1.00	0.75	1.00	0.025
		AG	11 (7.4)	9 (6.4)	1.16 (0.47–2.89)		0.00 (0.00–NA)	
*TLR9* − 1237T/C	Codominant	TT	146 (98.0)	140 (100.0)	1.00	0.14	1.00	0.24
		TC	2 (1.3)	0 (0.0)	NA (0.00–NA)		NA (0.00–NA)	
		CC	1 (0.7)	0 (0.0)	NA (0.00–NA)		0.00 (0.00–NA)	
	Dominant	TT	146 (98.0)	140 (100.0)	1.00	0.045	1.00	0.09
		TC-CC	3 (2.0)	0 (0.0)	NA (0.00–NA)		NA (0.00–NA)	
	Recessive	TT-TC	148 (99.3)	140 (0.0)	1.00	0.25	1.00	NA
		CC	1 (0.7)	0 (0.0)	NA (0.00–NA)		0.00 (0.00–NA)	
	Overdominant	TT-CC	147 (98.7)	140 (0.0)	1.00	0.1	1.00	0.09
		TC	2 (1.3)	0 (0.0)	NA (0.00–NA)		NA (0.00–NA)	
*TLR9* 1174G/A	Codominant	GG	77 (51.7)	88 (62.9)	1.00	**0.001**	1.00	0.028
		GA	70 (47.0)	42 (30.0)	1.90 (1.17–3.11)		2.29 (1.14–4.59)	
		AA	2 (1.3)	10 (7.1)	0.23 (0.05–1.08)		0.43 (0.05–3.52)	
	Dominant	GG	77 (51.7)	88 (62.9)	1.00	0.055	1.00	0.056
		GA-AA	72 (48.3)	52 (37.1)	1.58 (0.99–2.53)		1.94 (0.98–3.81)	
	Recessive	GG-GA	147 (98.7)	130 (92.9)	1.00	**0.01**	1.00	0.19
		AA	2 (1.3)	10 (7.1)	0.18 (0.04–0.82)		0.30 (0.04–2.43)	
	Overdominant	GG-AA	79 (53.0)	98 (70.0)	1.00	**0.003**	1.00	0.011
		GA	70 (47.0)	42 (30.0)	2.07 (1.27–3.35)		2.43 (1.22–4.83)	

The significance level after Bonferroni correction for multiple testing is 0.01 (raw *p*-value/5). Significant *p*-values were highlighted in bold.

*IM* infectious mononucleosis, *OR* odds ratio, *95% CI* 95% confidence interval, *p* logistic regression model, *NA* non-available.

^a^Values are the number of examined children (%).

^b^Adjusted analysis was carried out for EBV DNA copy number in whole-blood samples.

### Polymorphisms in *TLR* genes influence the risk of the IM symptoms

**Figure 1:**
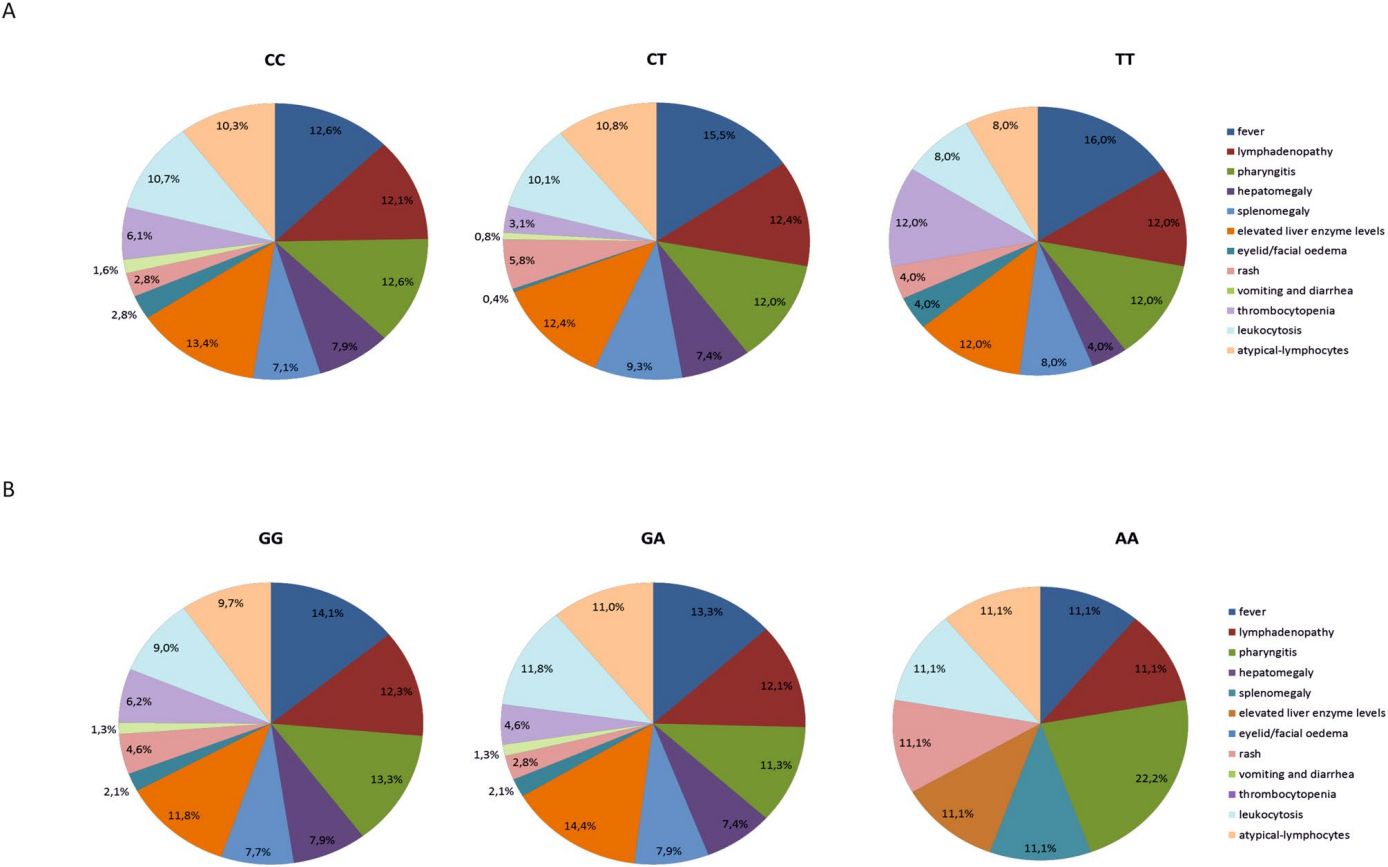
Pie charts show the percentage distribution of the symptoms of IM in pediatric patients with (**A**) *TLR2* 2029C/T and (**B**) *TLR9* 1174G/A SNPs.

The *TLR2* 2029 CC genotype was associated with elevated liver enzyme levels (OR 3.025; 95% CI 1.321–6.927; *p* = 0.009) and leukocytosis (OR 2.574; 95% CI 1.129–5.866; *p* = 0.024) (Table 4). In patients with this SNP, the liver enzyme levels ranged as follows: serum aspartate aminotransferase (AST), 18–467 IU/l; alanine aminotransferase (ALT), 11–907 IU/l; and gamma-glutamyl transpeptidase (GGT), 12–196 IU/l. In contrast, the heterozygous genotype of the *TLR2* 2029C/T SNP was detected in 50.0% (15/30) patients with rash and was associated with at least threefold increased risk of incidence of this finding (OR 3.435; 95% CI 1.363–8.660; *p* = 0.009). The carriers of the heterozygous variant of the *TLR4* 896 A/G SNP had at least tenfold higher risk of elevated ALT and AST levels (OR 10.000; 95% CI 1.122–89.113; *p* = 0.039 and OR 13.846; 95% CI 1.544–124.135; *p* = 0.019, respectively) (Table 4). This genotype was also associated with an almost sevenfold increased risk of thrombocytopenia (OR 6.909; 95% CI 1.325–36.035; *p* = 0.022), although this genotype was detected in a small number of patients (14.5%). For the *TLR9* 1174G/A SNP, the GA genotype was associated with higher risk of elevated liver enzyme levels and leukocytosis (OR 3.055; 95% CI 1.333–7.000; *p* = 0.008 and OR 2.412; 95% CI 1.143–5.088; *p* = 0.021, respectively), whereas the risk of these symptoms were decreased in patients with wild-type genotype (OR 0.370; 95% CI 0.164–0.836; *p* = 0.017 and OR 0.447; 95% CI 0.213–0.939; *p* = 0.034, respectively). Pie charts show the percentage distribution of the mononucleosis symptoms in patients with the *TLR2* 2029C/T and the *TLR9* 1174G/A SNPs (Fig. 1). Furthermore, coexistent mutations in the *TLR4* (896 A/G SNP) and the *TLR9* (117G/A SNP) genes were detected in 11/104 (10.6%) of IM patients and were associated with an almost a sevenfold higher risk of elevated AST level (OR 6.827; 95% CI 1.247–37.380; *p* = 0.027, unadjusted model; OR 8.750; 95% CI 1.331–57.528; *p* = 0.024, adjusted model). A tendency towards a higher risk of occurrence of atypical-lymphocytes in patients with mutation in both SNPs was also observed (OR 7.420; 95% CI 0.881–62.477; *p* = 0.065, unadjusted model; OR 8.272; 95% CI 0.887–77.130; *p* = 0.044, adjusted model). No other significant correlation between coexisting SNPs and IM symptoms or viral load were found (*p* > 0.05).

**Table 4:** *TLR* SNPs as prognostic factors for the risk of symptoms/signs of EBV-related infectious mononucleosis.

*TLR* SNP	Genotype	Symptom/sign	n (%)^a^	Unadjusted		Adjusted^b^	
				OR (95% CI)	*p*-value	OR (95% CI)	*p*-value
*TLR2* 2029C/T	CC	Rash	14/30 (46.7)	0.348 (0.142–0.853)	**0.021**	0.348 (0.138–0.880)	**0.026**
		Elevated liver enzyme level^c^	68/103 (66.0)	3.025 (1.321–6.927)	**0.009**	2.501 (1.035–6.042)	**0.042**
		Leukocytosis	54/82 (65.9)	2.574 (1.129–5.866)	**0.024**	2.716 (1.122–6.571)	**0.027**
	CT	Elevated liver enzyme level	32/103 (31.1)	0.356 (0.149–0.846)	**0.019**	0.419 (0.165–1.064)	0.067
		Rash	15/30 (50.0)	3.435 (1.363–8.660)	**0.009**	3.690 (1.407–9.678)	**0.008**
*TLR4* 896A/G	AG	ALT	8/62 (12.9)	10.000 (1.122–89.113)	**0.039**	9.025 (0.856–95.149)	0.067
		AST	8/55 (14.5)	13.846 (1.544–124.135)	**0.019**	14.498(1.378–152.558)	**0.026**
		Thrombocytopenia	6/42 (14.3)	6.909 (1.325–36.035)	**0.022**	5.822 (0.962–35.217)	0.055
	AA	Leukocytosis	67/103 (65.0)	0.290 (0.088–0.961)	**0.043**	0.301 (0.088–1.034)	0.056
		Thrombocytopenia	33/42 (78.6)	0.329 (0.112–0.964)	**0.043**	0.326 (0.098–1.079)	0.067
*TLR9* 1174G/A	GG	Elevated liver enzyme level	46/103 (44.7)	0.370 (0.164–0.836)	**0.017**	0.286 (0.117–0.697)	**0.006**
		Leukocytosis	35/82 (42.7)	0.447 (0.213–0.939)	**0.034**	0.434 (0.200–0.941)	**0.035**
	GA	Elevated liver enzyme level	56/103 (54.4)	3.055 (1.333–7.000)	**0.008**	3.986 (1.606–9.892)	**0.003**
		Atypical-lymphocytes	43/82 (52.4)	2.115 (0.989–4.524)	0.054	2.311 (1.014–5.268)	**0.046**
		Leukocytosis	46/82 (56.1)	2.412 (1.143–5.088)	**0.021**	2.469 (1.134–5.375)	**0.023**

Significant *p*-values were highlighted in bold.

*OR* odds ratio, *95% CI* 95% confidence interval, *ALT* alanine aminotransferase, *AST* aspartate aminotransferase, *GGT* gamma-glutamyl transpeptidase.

^a^Values are the number of pediatric patients with specific symptom and the selected polymorphism/number of overall patients with the selected symptom (%).

^b^Adjusted analysis was carried out for EBV DNA copy number in whole-blood samples and patient age.

^c^Elevated enzyme level, including ALT, AST and GGT.

### The wild-type genotype of the *TLR2* 2029C/T polymorphism is associated with the EBV replication

**Figure 2:**
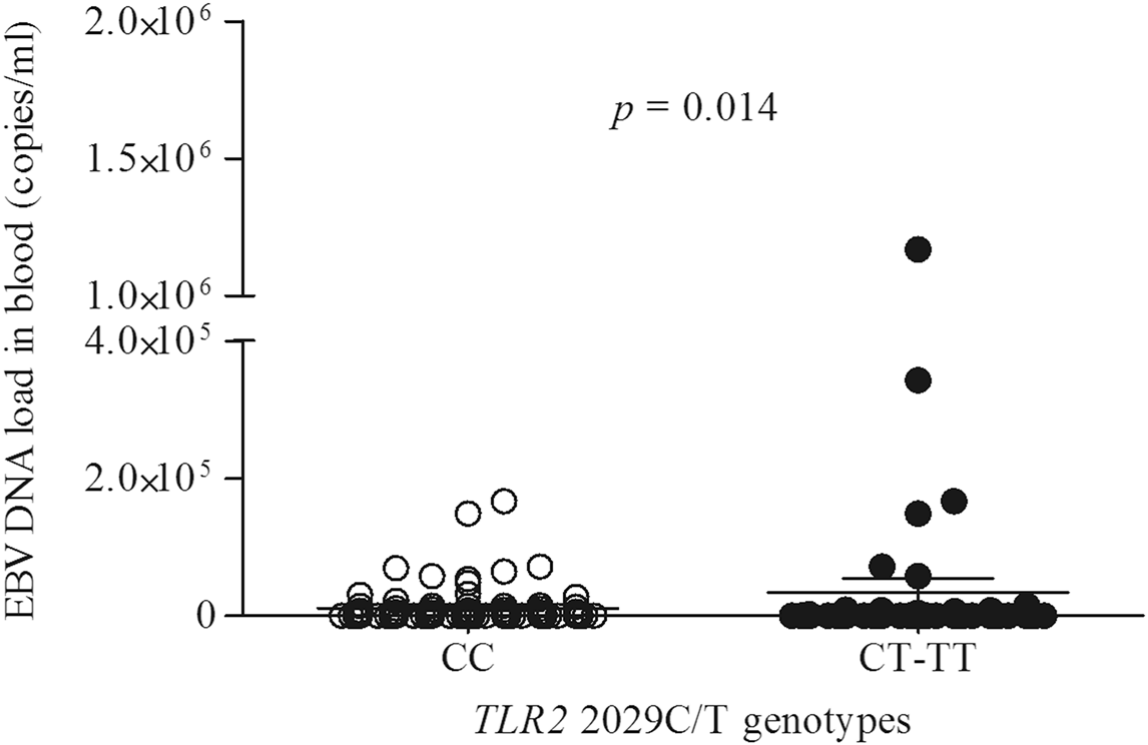
The EBV DNAemia level among IM patients with *TLR2* 2029C/T SNP (*n* = 149). Bars represent the mean values and standard error of the mean (mean ± SEM) of the viral load. *p*-value via a Mann–Whitney U test.

To further examine the association between the *TLR* polymorphisms and viral infection, we correlated the specific *TLR* SNPs with the EBV DNA levels in the peripheral blood of IM patients. As shown in Fig. 2, the median EBV DNA levels in blood were lower among individuals who had a wild-type genotype for the *TLR2* 2029C/T (mean 1.09 × 10^4^ copies/ml ± 2.66 × 10^4^ copies/ml) compared with those who were heterozygous or homozygous recessive (mean 3.40 × 10^4^ copies/ml ± 1.58 × 10^5^ copies/ml) for this polymorphism (*p* = 0.014). Age group-specific analyses confirmed that children carrying the wild-type genotype for the *TLR2* 2029C/T SNP had lower EBV DNAemia than those with heterozygous or homozygous recessive genotypes (mean 1.37 × 10^4^ copies/ml ± 3.24 × 10^4^ copies/ml vs. mean 3.21 × 10^4^ copies/ml ± 1.9 × 10^5^ copies/ml; *p* = 0.015). Such a correlation was observed also for adolescents (mean 7.81 × 10^3^ copies/ml ± 1.59 × 10^4^ copies/ml vs. mean 2.07 × 10^4^ copies/ml ± 8.05 × 10^4^ copies/ml; *p* = 0.042, respectively). No association was observed between the EBV DNAemia and any other *TLR* polymorphisms (*p* > 0.05) in both study groups.

### *TLR* haplotypes in patients with IM

Haplotype analysis of the *TLR2* 2029C/T, 2258G/A, the *TLR4* 896A/G, and the *TLR9* − 1237T/C, and 1174G/A SNPs showed that the most frequent haplotype was CGATG (53.5%), which was detected in 48.9% of patients with IM and 58.1% of healthy individuals. The CAATA haplotype was detected at a minor frequency in 1.5% of patients with IM and 2.0% of healthy volunteers, whereas haplotype TAATG was determined only in EBV-infected patients (3.8%). We found no evidence for linkage disequilibrium (LD) for the all examined *TLR* SNPs (*p* > 0.05, r^2^ < 0.2).

## Discussion

TLRs have been demonstrated to play a crucial role in modulating innate recognition of viruses not only by serving as pathogen sensors but also by activating signaling pathways that result in the increased production of proinflammatory cytokines and type I IFNs. This preliminary study provides the first evidence that *TLR* polymorphisms seem to influence the outcome of the infectious mononucleosis in children and adolescents. Patients with the *TLR4* 896A/G and the *TLR9* 1174G/A polymorphisms had a higher risk of IM and significantly increased the frequency of specific manifestations when compared with subjects with the wild-type genotype.

No studies were examining the allele frequency distribution and the role of *TLR* SNPs in patients with EBV-related IM. We observed that the *TLR4* 896A/G SNP and an intronic polymorphism 1174G/A in exon 2 of the *TLR9* gene were significantly associated with increased risk of EBV infection in our population. Polymorphisms in *TLR4* gene, which is located in chromosome 9q33.1, has been implicated so far in the pathogenesis of several infectious diseases, including bacterial^23–25^, fungal^26^, parasitic^27^, and some viral infection, such as human immunodeficiency virus^28,29^, Kaposi sarcoma-associated herpesvirus^30^, and respiratory syncytial virus (RSV)^31^. It was previously described that missense mutations and single nucleotide polymorphisms in the *TLR4* gene could alter the function of the receptor and impairs recognition of the pathogens^30,32^. The aspartic acid to glycine substitution at residue 299 polymorphism (896A/G) of the *TLR4* was found to be associated with susceptibility to bacterial pneumonia, possibly through impaired first-line defense mechanisms^33^. Individuals carrying the *TLR4* 896A/G polymorphisms were significantly less responsive to bacterial peptides derived from *Escherichia coli* and *Porphyromonas gingivalis* compared with the wild-type subjects^34,35^. Functional studies have demonstrated that this SNP was also associated with impaired NF-κB and IFN regulatory factor 3 activation in response to lipopolysaccharide, F protein from RSV, and chlamydial Hsp60^36^. It was found that TLR9 contributes to the recognition of EBV and is expressed on B cells, a natural target of the virus infection^18,37^. The *TLR9* 1174 G/A SNP was linked to rapid disease progression in children with malaria and HIV-infected patients^38,39^ and was also associated with nonresponse to anti-TNF treatment among patients with inflammatory bowel disease^40^. In our previous studies, no significant differences in the frequencies of these polymorphisms were detected among CMV-infected and healthy infants within the same ethnic group^20,41^. However, a reduced risk of the CMV infection in infants with the 1174 GG genotype was noticed^20^. We suggest that both the *TLR4* 896A/G and the *TLR9* 1174 G/A polymorphisms may influence the immune response and impair NF-κB activation in EBV infection.

Several studies have implicated that *TLR* polymorphisms are involved in the development of lymphoid and non-lymphoid EBV-associated malignancies^42–50^. It has been previously reported that the − 16933T/A SNP in the *TLR2* gene increased the risk of follicular lymphoma and decreased the risk of chronic lymphocytic leukemia^43^. Mutation present in both alleles of the *TLR2* 2029C/T and 2258G/A SNPs were associated with susceptibility to gastric carcinoma in China^42^. In the *TLR4* gene, the 896A/G polymorphism influences the risk of mucosa-associated lymphoid tissue lymphoma and Hodgkin lymphoma^42^, but not with non-Hodgkin lymphoma^45,46^. The combined CT/TT genotype of the 1196C/T SNP and the GC genotype of the 11350G/C polymorphism in the 3′-untranslated region of the *TLR4* gene may alter its expression, influence the expression of inflammatory cytokines and chemokines, and increased the risk of nasopharyngeal carcinoma (NPC)^49,50^. The occurrence and progression of NPC were also associated with the *TLR9* − 1486T/C SNP^48^. The *TLR9* − 1486 CC genotype increased the susceptibility of NPC in the Chinese population and patients with this genotype were inclined to advanced tumor stage and lymph node metastasis. No association between the *TLR9* − 1237T/C and 2848C/T SNPs and the risk of NPC was found^48^. Nevertheless, carriers of the − 1237 C and 2848 A allele had been reported to be associated with an increased risk of Hodgkin’s lymphoma in the Caucasian population^47^. The − 1237T/C polymorphism was associated with non-Hodgkin lymphoma in Portuguese and Italian, but not in the United States cohort of patients^44^. Analysis in silico of the *TLR9* promoter showed that the mutated − 1237T/C and − 1486T/C variants create a putative c-Rel/NF-kB transcription factor binding sites that influence transcriptional regulation of the gene^51^. The NF-kB binding site in the C allele at the position − 1237 enhanced the transcriptional activity of the *TLR9* gene and affect activation of proinflammatory cytokines, chemokines, and the adaptive immune response^52^. In contrast, another study revealed that the TT allelic variant of − 1237T/C SNP had higher transcriptional activity^53^.

A few investigations indicated that the risk of developing infectious mononucleosis after primary infection with EBV correlates with the age of patients and adolescents would react more strongly against EBV infection than children^54–56^. It is also known that the frequency of IM symptoms depends on age, ethnicity and geographic location^57–59^. Most people are exposed to the virus as children, when the disease produces flu-like symptoms such as pharyngitis, whereas a characteristic triad of fever, pharyngitis, and lymphadenopathy is more frequently presented in adolescents and young adults. In this study, we observed that elevated liver enzyme levels, thrombocytopenia, as well as higher atypical-lymphocytes were more frequent in adolescents than children. This result supports the hypothesis that the prevalence of acute IM rises significantly with patient age, and is common in adolescents^54,55^. These findings are coincident with that described by Wang et al., who demonstrated that young patients had significantly increased levels of liver enzymes and atypical-lymphocytes, while no significant difference in the prevalence of fever between children and adolescents was observed^58^. The most significant laboratory abnormality found in the present study was atypical-lymphocytes, which occurred more frequently in adolescents than in children (66.1% *vs.* 48.5%). The low platelet count, in course of IM, was observed in 26.3% adult patients in France (age 16–53 years)^59^, less than 1% young adults in the United States^60^, 2.3–4.5% preschool children and youth patients in China^58,61^, and 7.3% Mexican children (age 0–17.5 years)^62^. The higher incidence of rash in children than in adolescents (25.8% vs. 10.7%) was observed in our study. Almost one-third population of Israeli patients up to 18 years had a rash during the IM, but the age of the patients was not associated with the development of this symptom^57^. Similarly, no statistically significant difference in patient age and the incidence of rash was found among preschool children (21.5%) and youth patients (13.9%) in Beijing, China^58^. Part of the explanation that would elucidate the more frequent risk of IM in adolescents and young adults than in children is that the percentage of CD8+ T cell counts increased and CD4+ T cell counts decreased with age increment^61^. Moreover, significantly higher levels of early-differentiated CD56^dim^ NKG2A^+^ killer-cell immunoglobulin-like receptors (KIR)^−^ NK cells in the peripheral blood of children than in adolescents and young adults may affect the course of EBV infection^63,64^. It was also found that polymorphisms in the HLA class I locus may predispose patients to the development of IM upon primary EBV infection^65^. McAulay et al. described that the nature of primary EBV infection and the level of viral persistence can be determined by the genetic variation in T cell responses^65^. It is also possible that adolescents receive a larger amount of the virus through deep kissing, while young children probably acquire EBV from parents or guardians, who during reactivation of infection transmit smaller infectious amounts of EBV, or from siblings or other children^54,66,67^. Furthermore, the heterophile antibody test is less sensitive and often unreliable in young children^3,56,68^. It is also known that EBV VCAs cause lifelong persistent IgG titers, while antibodies of the IgM type are produced only transiently but are not necessarily produced in all patients with primary infections^69^. However, to understand the differential impact age on susceptibility to primary EBV infection, further research in age-matched patient groups are needed.

The present study is first to examine the distribution and possible association between the *TLR* gene polymorphisms and the clinical and/or laboratory findings of infectious mononucleosis. Similar to other genetic association studies, this study has some potential limitations. First, the present study may be limited by a relatively small number of participants. However, we enrolled patients from a well-characterized population and all noticeable symptoms were verified by experienced clinicians. Moreover, serological documentation of EBV infection indicates the higher incidence of advanced-stage disease or the delayed recognition of IM in the examined children than adolescents. Further studies in a larger sample group are needed to confirm our findings and to evaluate the function of the disease-associated *TLR* polymorphisms.

## Methods

### Study population

A total of 289 blood samples was obtained from 149 patients with IM (median age: 8.2 years; range 3 months–17.5 years) and 140 unrelated seronegative and aviremic healthy volunteers, without clinical symptoms/signs of EBV infection. Whole mouth saliva and throat swabs samples from patients with IM were also collected for medical research. The subjects involved in the study were recruited from the Department of Pediatric Infectious Diseases, Wroclaw Medical University in Wroclaw (between April 2014 and August 2018) and Polish Mother’s Memorial Hospital Research Institute in Lodz (between September 2011 and April 2018). The presence of EBNA-IgG, EBV-IgM, and EBV-IgG or VCA-IgG was measured using an immunochemiluminescence assay (CLIA, DiaSorin, Saluggia, Italy) according to the manufacturer’s instructions. The presence of EBV DNA in the whole blood and/or the presence of IgM antibodies to VCA in the absence of measurable antibodies to EBNA-1 was considered the evidence for primary EBV infection as described previously^3,70^. AST, ALT, and GGT were determined by dry chemistry method using Johnson & Johnson Vitros 5.1 FS Chemistry Analyzer (Ortho Clinical Diagnostics, Raritan, NJ, USA) or by the kinetic method using Architect 2000 (Abbott Laboratories, Abbott Park, IL, USA). Mild elevations of ALT and AST were commonly discovered in individuals with mild or no symptoms and were defined as increased liver enzyme values higher than 2–5 times the upper limit of normal. Liver disease was diagnosed in patients with elevated liver enzyme levels greater than 5 times the upper limit of the reference range. Platelet counts were tested using optical and/or impedance methods using the Sysmex XE-2000 Analyzer (Sysmex Corp., Kobe, Japan). Thrombocytopenia was recognized when the platelet count fell below 150 × 10^9^/l of blood, whereas leukocytosis was classified as elevated white blood cells count above the age-specific reference values. All the individuals with EBV infection were ethnically classified as European descents and were enrolled from the Central and South-West areas of Poland. Clinical data from EBV infected patients were summarized in Table 1. This study was approved by the appropriate Bioethics Committee of the Medical University of Lodz (RNN/120/09/KE) and the Ethics Committee of the Polish Mother’s Memorial Hospital Research Institute (7/2014). Written informed consent was provided from parents of all children who participated in the study. All experiments were performed in accordance with relevant guidelines and regulations.

### Genotyping of *TLRs* polymorphisms

Total genomic DNA was extracted from the peripheral blood, whole mouth saliva, and throat swabs samples using the QIAamp DNA Blood Mini Kit (Qiagen GmbH, Hilden, Germany) according to the manufacturer’s instruction. Molecular typing of *TLR2* (2029C/T, rs121917864; 2258G/A, rs5743708), *TLR4* (896A/G, rs4986790) and *TLR9* (− 1237T/C, rs5743836; − 1486T/C, rs187084; 1174G/A, rs352139; and 2848C/T, rs352140) SNPs were performed by polymerase chain reaction–restriction fragment length polymorphism (PCR–RFLP) as described elsewhere^20,41^. The results were confirmed by the sequencing of randomly selected samples of each *TLR* gene using the BigDye Terminator v3.1 Cycle Sequencing Kit and the 96-capillary 3730xl DNA Analyzer (Applied Biosystems).

### Assessment of EBV replication

EBV DNA copy numbers in clinical samples were determined using a 7900HT Fast Real-Time PCR system (Applied Biosystems, Foster City, CA, USA) with a commercially available Epstein Barr Virus (Human Herpes virus 4) nonglycosylated membrane protein (*BNRF1*) gene Kit (Primerdesign Ltd., York House, UK) according to the manufacturer’s instruction. The PCR conditions were as follows: 2 min at 95 °C, followed by 50 cycles of 95 °C for 10 s and 60 °C for 60 s. A negative control without template DNA was included in every amplification run. The sensitivity of the assay has been determined to be 200 copies/ml of whole blood, mouth saliva, and throat swabs.

### Statistical analysis

Data were statistically analyzed using the GraphPad Prism 5.00 (GraphPad Software, San Diego, CA, USA) and SPSS statistical software package for Windows 25.0 (SPSS, Chicago, IL, USA). Categorical data were analyzed by the Chi-square test and Fisher’s exact test, while the Mann–Whitney U test was used to estimate the association between *TLR* SNPs and the viral load. The association of polymorphisms and clinical characteristics of the patients was estimated by odds ratio (OR) with a 95% confidence interval (95% CI). A *p*-value of less than 0.05 was considered statistically significant. The HWE, LD, and haplotype analyses were performed using the SNPStats software (https://www.snpstats.net/start.htm). The Bonferroni correction of the significance level was applied for five multiple comparisons; the significance level for *p*_*B*_ is 0.01 instead standard 0.05.
